# The p52-ZER6/G6PD axis alters aerobic glycolysis and promotes tumor progression by activating the pentose phosphate pathway

**DOI:** 10.1038/s41389-023-00464-4

**Published:** 2023-03-28

**Authors:** Yu Tang, Wenfang Li, Li Qiu, Xia Zhang, Lei Zhang, Makoto Miyagishi, Hezhao Zhao, Shourong Wu, Vivi Kasim

**Affiliations:** 1 Key Laboratory of Biorheological Science and Technology, Ministry of Education, College of Bioengineering, Chongqing University, Chongqing, 400044 China; 2 The 111 Project Laboratory of Biomechanics and Tissue Repair, College of Bioengineering, Chongqing University, Chongqing, 400044 China; 3 Molecular Composite Medicine Research Group, Biomedical Research Institute, National Institute of Advanced Industrial Science and Technology (AIST), Tsukuba, 305-8566 Japan; 4 Department of Gastrointestinal Surgery, Chongqing University Cancer Hospital, Chongqing University, Chongqing, 400030 China; 5 Chongqing Key Laboratory of Translational Research for Cancer Metastasis and Individualized Treatment, Chongqing University Cancer Hospital, Chongqing University, Chongqing, 400030 China

**Keywords:** Cancer metabolism, Colorectal cancer

## Abstract

Abnormal glucose metabolism is a highlight of tumor metabolic reprogramming and is closely related to the development of malignancies. p52-ZER6, a C_2_H_2_-type zinc finger protein, promotes cell proliferation and tumorigenesis. However, its role in the regulation of biological and pathological functions remains poorly understood. Here, we examined the role of p52-ZER6 in tumor cell metabolic reprogramming. Specifically, we demonstrated that p52-ZER6 promotes tumor glucose metabolic reprogramming by positively regulating the transcription of *glucose-6-phosphate dehydrogenase* (*G6PD*), the rate-limiting enzyme in the pentose phosphate pathway (PPP). By activating the PPP, p52-ZER6 was found to enhance the production of nucleotides and nicotinamide adenine dinucleotide phosphate, thereby providing tumor cells with the building blocks of ribonucleic acids and cellular reductants for reactive oxygen species scavenging, which subsequently promotes tumor cell proliferation and viability. Importantly, p52-ZER6 promoted PPP-mediated tumorigenesis in a p53-independent manner. Taken together, these findings reveal a novel role for p52-ZER6 in regulating *G6PD* transcription via a p53-independent process, ultimately resulting in tumor cell metabolic reprogramming and tumorigenesis. Our results suggest that *p52-ZER6* is a potential target for the diagnosis and treatment of tumors and metabolic disorders.

## Introduction

Tumor metabolic reprogramming is a hallmark of malignancy and plays a crucial role in meeting the cell’s biogenesis demands and rapid proliferation [1]. Furthermore, it facilitates tumor cell survival in response to certain genetic and environmental stressors. Glucose metabolic reprogramming, known also as the Warburg effect, occurs when tumor cells increase their glycolytic rate and exhibit a preference for glycolysis followed by lactate fermentation, instead of oxidative phosphorylation, even in the presence of sufficient oxygen [2–4]. This phenomenon favors various branch reactions that yield glycolytic intermediates for downstream anabolic processes. One such branch is the pentose phosphate pathway (PPP) involved in glucose catabolism. The PPP is critical for tumor cell proliferation, as well as for maintaining the redox balance under stress conditions [5]. Furthermore, it produces ribose-5-phosphate, a building block for ribonucleotides essential in DNA synthesis, and provides nicotinamide adenine dinucleotide phosphate (NADPH), a cellular reductant important for lipid biosynthesis and for maintaining cellular redox homeostasis by scavenging reactive oxygen species (ROS) [5–7]. Due to its critical role in tumorigenesis, targeting glucose-6-phosphate dehydrogenase (G6PD), the first rate-limiting enzyme in the PPP, has attracted attention as a potential strategy for treating tumors [8–10], even though the regulatory mechanism controlling the PPP is not fully understood.

Zinc-finger estrogen receptor interaction clone 6 (ZER6) is a transcription factor that contains six Cys_2_His_2_ zinc-finger domains. ZER6 can be spliced into two isoforms with similar carboxyl termini but different amino termini, namely p52-ZER6 and p71-ZER6 [11]. p71-ZER6 has a HUB-1 domain and a full-length KRAB domain; whereas p52-ZER6 does not contain the HUB-1 domain and possesses only a truncated KRAB domain. Even though it was discovered 20 years ago, its biological and pathological functions remain unknown, and only recently, p52-ZER6 was identified as an oncogene that could enhance tumorigenesis by promoting ubiquitination/proteasomal degradation of tumor suppressor p53 [12]. Furthermore, while various C_2_H_2_ zinc finger proteins could regulate tumor metabolic reprogramming [13, 14], whether ZER6 is involved in this process remains elusive.

In this study, we aimed to determine the role of p52-ZER6 in the metabolic reprogramming of tumor cells. We identified G6PD as a novel target of p52-ZER6. Through in vitro and in vivo experiments, we found that p52-ZER6 could activate *G6PD* transcription, leading to an increase in PPP activity and, in turn, tumorigenesis. Furthermore, we show that p52-ZER6-mediated regulation of *G6PD* occurs in a p53-independent manner, most plausibly through direct binding of p52-ZER6 to the *G6PD* promoter. Hence, this study reveals p52-ZER6 as a novel regulator of the PPP and, thereby, tumor progression. Importantly, this study points to p52-ZER6 as a new target for anti-tumor therapeutic strategies.

## Materials and methods

### Vectors construction

shRNA expression vectors targeting *ZER6*, *p52-ZER6*, and *p71-ZER6*, as well as *p52-ZER6* overexpression vector were constructed as described previously [12]. The target sequences specific for *ZER6*, *p52-ZER6* and *p71-ZER6* are as follow: shZER6-1: 5′-GCT AGA GTC TGG ACT TAT A-3′, shZER6-2: 5′-ACT AGA GGG GGA ATA TAA T-3′, shp52-ZER6-1: 5′-GAG GGG ATG TGG TCT TGC T-3′, shp52-ZER6-2: 5′-GAT TGA GAG GTA GCG GGA A-3′, shp71-ZER6-1: 5′-GGG GGA GGT AGG GCT GGG T-3′, shp71-ZER6-2: 5′-GCT TGG GGC TGG GCG TGG T-3′.

*G6PD* overexpression vector (pcG6PD) was constructed as described previously [15]. Briefly, total RNA extracted from HCT116 cells was reverse-transcribed using PrimeScript Reagent Kit (Takara Bio, Dalian, China) with gDNA eraser to obtain cDNA. The corresponding region was then amplified from the cDNA using the Takara Prime STAR Max DNA Polymerase (Takara Bio), and the amplicon was then inserted into the pcEF9-Puro vector bringing puromycin resistance gene.

For G6PD luciferase reporter vectors, the −2289 to +622 (G6PD-luc-1), the −1657 to +622 (G6PD-luc-2), the −1,158 to +622 (G6PD-luc-3), and the +70 to +622 (G6PD-luc-4) regions of the *G6PD* promoter were cloned into the *Nhe*I and *Stu*I sites of the pGL4.13 vector (Promega, Madison, WI). Human genome DNA extracted from wild-type HCT116 cells using TIANamp Genomic DNA Kit (Tiangen Biotech, Beijing, China) was used as template for amplifying the promoter regions. G6PD-luciferase vector with mutated p52-ZER6 binding site (G6PD^mut^-luc) was constructed using a Site-directed Gene Mutagenesis Kit (Beyotime Biotechnology, Shanghai, China) based on the site-specific mutagenesis method.

### Cell lines and cell culture

Wild-type HCT116, HCC-LM3, and MCF-7 cell lines were purchased from the Cell Bank of Chinese Academy of Sciences (Shanghai, China), and the HCT116^p53null^ cell line was kindly provided by Dr. Bert Vogelstein at John Hopkins University School of Medicine. Wild-type HCT116 and HCT116^p53null^ cells were maintained in McCoy’s 5 A medium (Gibco, Grand Island, NY), while HCC-LM3 and MCF-7 cells were maintained in Dulbecco’s modified Eagle’s medium (Gibco). Cells were cultured with 10% fetal bovine serum (Biological Industries, Beit Haemek, Israel) and 1% penicillin-streptomycin. All cell lines were verified using short-tandem repeat profiling method, and have been routinely tested every 6 months for mycoplasma contamination using Mycoplasma Detection Kit-Quick Test (Biotool, Houston, TX). Transfection was performed using Lipofectamine 2000 (Invitrogen Life Technologies, Carlsbad, CA) according to the manufacturer’s instruction.

For gene silencing and gene overexpression experiments, cells were seeded in 6-well plates and transfected with 2 μg of the indicated vectors. 24 h later, cells were subjected to puromycin selection (final concentration: 1 μg/ml) for 36 h to eliminate untransfected cells. For rescue experiments, cells were transfected with 1 μg of shp52-ZER6 and 1 μg of pcG6PD vectors and subjected to puromycin selection to eliminate untransfected cells.

For establishing *p52-ZER6*-silenced HCT116^p53null^ cells (HCT116^p53null^/shp52-ZER6 cells) and *p52-ZER6*-silenced, *G6PD*-overexpressed HCT116^p53null^ cells (HCT116^p53null^/shp52-ZER6/pcG6PD cells) stable cell lines, cells were seeded in 10 cm well plates, and transfected with 12 μg of shCon or shp52-ZER6 and 6 μg of pcCon or pcG6PD vectors. Stable cell lines were established by performing puromycin selection.

### Clinical human colorectal carcinoma specimens

Human colorectal carcinoma fresh specimens were obtained from patients undergoing surgery at Chongqing University Cancer Hospital (Chongqing, China). Patients did not receive chemotherapy, radiotherapy, or other adjuvant therapies prior to the surgery. The specimens were snap-frozen in liquid nitrogen. Prior patients’ written informed consents were obtained. The study was approved by the Institutional Research Ethics Committee of Chongqing University Cancer Hospital, and was conducted in accordance with Declaration of Helsinki.

### Animal experiment

For the in vivo tumor study, BALB/c-nu/nu mice (male; body weight, 18–22 g; 6 weeks old) were purchased from Chongqing Medical University (Chongqing, China). Animal study was approved by the Institutional Ethics Committee of Chongqing Medical University, and carried out at the Chongqing Medical University. All animal experiments conformed to the approved guidelines of the Animal Care and Use Committee of Chongqing Medical University. All efforts were made to minimize suffering. To generate an experimental subcutaneous tumor model, BALB/c-nu/nu mice were randomly divided into three groups (*n* = 6), and each group was injected subcutaneously with 5×10^6^ indicated stable cells. Tumor size (V) was evaluated by a caliper every 2 days with reference to the following equation: V = a × b^2^/2, where a and b are the major and minor axes of the tumor, respectively. The investigator was blinded to the group allocation and during the assessment.

### in situ hybridization assay

For in situ hybridization assay, tissue sections were rehydrated and digested with protease K for 25 min at 37 °C. After pre-hybridization at 37 °C for 1 h, hybridization was carried out by overnight incubation at 37 °C with 8 ng/μl digoxin-conjugated probe specific for the p52-ZER6 isoform (91–113 at the 5’ UTR of p52-ZER6 mRNA: TCT CGT CTT CGA CCG CAT CCC TC). After being washed with SSC washing buffer at 37 °C, blocking was performed with bovine serum albumin (BSA, final concentration: 3%) for 30 min. Specifically bound probes were detected by anti-DIG-HRP antibody (dilution: 1/400; Jackson Immunoresearch, West Grove, PA). Nuclei were stained with hematoxylin. Images were taken using Pannoramic Midi (3DHistech, Budapest, Hungary).

### ROS staining

For cellular experiments, cells were transfected with indicated shRNA expression vectors or overexpression vector and selected using puromycin as indicated above. After puromycin selection, the fluorescent probe DCFH-DA was loaded into the cells using Reactive Oxygen Species Assay Kit (Beyotime Biotechnology, Shanghai, China). Intracellular ROS level was determined using flow cytometry. For tissue experiments, frozen sections were rewarmed at room temperature. Tissue sections were then incubated with dihydroethidium (DHE, final concentration: 2 mg/ml; Sigma Aldrich, St. Louis, MO) for 30 min at 37 °C to stain the ROS. Nuclei were stained with DAPI for 15 min. Images were taken using Pannoramic Midi (3DHistech).

### Western blotting and quantitative real time-PCR (qRT-PCR) analysis

Detailed methods for western blotting and qRT-PCR analysis are described in the Supplementary Materials and Methods. The sequences of the primers and antibodies used are shown in the Supplementary Tables S1 and S2, respectively.

### Statistical analysis

All quantification results were presented as mean ± SD (*n* = 3, unless otherwise indicated). Statistical analysis was performed using two-tailed unpaired Student’s *t*-test conducted using GraphPad Prism 8.0. When more than two groups were compared, one-way ANOVA analyses were performed. A value of *P < 0.05 was considered statistically significant.

## Results

### p52-ZER6 enhances G6PD expression in tumor cells

**Fig. 1:**
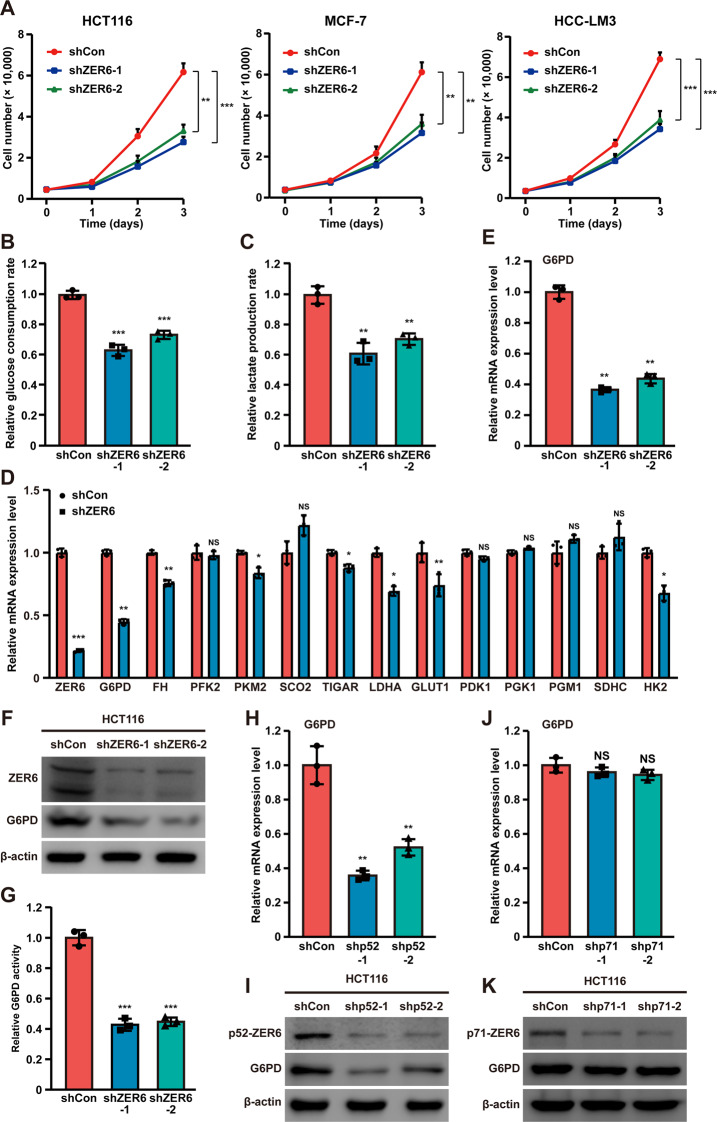
ZER6 alters tumor cells glucose metabolism. **A** Viability of *ZER6*-silenced HCT116, MCF-7 and HCC-LM3 cells at indicated time points. **B**, **C** Glucose consumption (**B**) and lactate production (**C**) levels in *ZER6*-silenced HCT116 cells. **D** mRNA expression levels of glucose metabolism-related genes in *ZER6*-silenced HCT116 cells, as analyzed using quantitative real-time PCR (qRT-PCR). **E**, **F** G6PD mRNA (**E**) and protein (**F**) expression levels in *ZER6*-silenced HCT116 cells, as analyzed using qRT-PCR and western blotting, respectively. **G** G6PD enzymatic activity in *ZER6*-silenced HCT116 cells. **H, I** G6PD mRNA (**H**) and protein (**I**) expression levels in *p52-ZER6*-silenced HCT116 cells, as analyzed using qRT-PCR and western blotting, respectively. **J**, **K** G6PD mRNA (**J**) and protein (**K**) expression levels in *p71-ZER6*-silenced HCT116 cells, as analyzed using qRT-PCR and western blotting, respectively. Cells transfected with shCon were used as controls. β-actin was used for qRT-PCR normalization and as western blotting loading control. Total protein was used for normalizing the levels of glucose consumption, lactate production, and G6PD enzymatic activity. Quantification data are expressed as mean ± SD (*n* = 3). shZER6: shRNA expression vector targeting *ZER6*; shp52: shRNA expression vector targeting *p52-ZER6*; shp71: shRNA expression vector targeting *p71-ZER6*; *P < 0.05; **P < 0.01; ***P < 0.001; NS not significant.

To explore the role of ZER6 in tumor cell metabolism, we constructed two shRNA vectors targeting different sites of *ZER6* (Figs. S1A–S1C). As shown in Fig. 1A, *ZER6* silencing significantly reduced cell numbers in colorectal carcinoma, breast cancer and hepatocarcinoma cell lines (HCT116, MCF-7 and HCC-LM3, respectively). Given that glucose metabolism is vital for highly proliferative tumor cells, we examined the effect of ZER6 on glucose consumption and lactate production. *ZER6* silencing significantly reduced both parameters (Figs. 1B, 1C), indicating that ZER6 enhances glucose metabolism in tumor cells.

To elucidate the molecular mechanism through which ZER6 regulates tumor cell glucose metabolism, we examined the effect of knocking down ZER6 on various glucose metabolism-related genes. *G6PD* was the most significantly suppressed gene in *ZER6*-silenced HCT116 cells (Fig. 1D). This finding was further confirmed at the mRNA and protein levels using two shRNA vectors targeting different *G6PD* sites (Figs. 1E, 1F), as well as with respect to G6PD enzymatic activity (Fig. 1G). Taken together, these results show that ZER6 regulates G6PD expression.

As reported previously, ZER6 exists as two isoforms, p52-ZER6 and p71-ZER6 (Fig. S2A). To explore which isoform regulated G6PD, we constructed two shRNA vectors targeting different sites on *p52-ZER6* and *p71-ZER6* (Fig. S2B) and examined their efficacies (Figs. S2C–S2F). Knocking down *p52-ZER6*, but not *p71-ZER6*, significantly suppressed G6PD mRNA and protein levels (Figs. 1H, 1I); whereas knocking down *p71-ZER6* had no significant effect on G6PD expression (Figs. 1J, 1K). These results indicate that ZER6-mediated regulation of G6PD is specific to p52-ZER6.

**Fig. 2:**
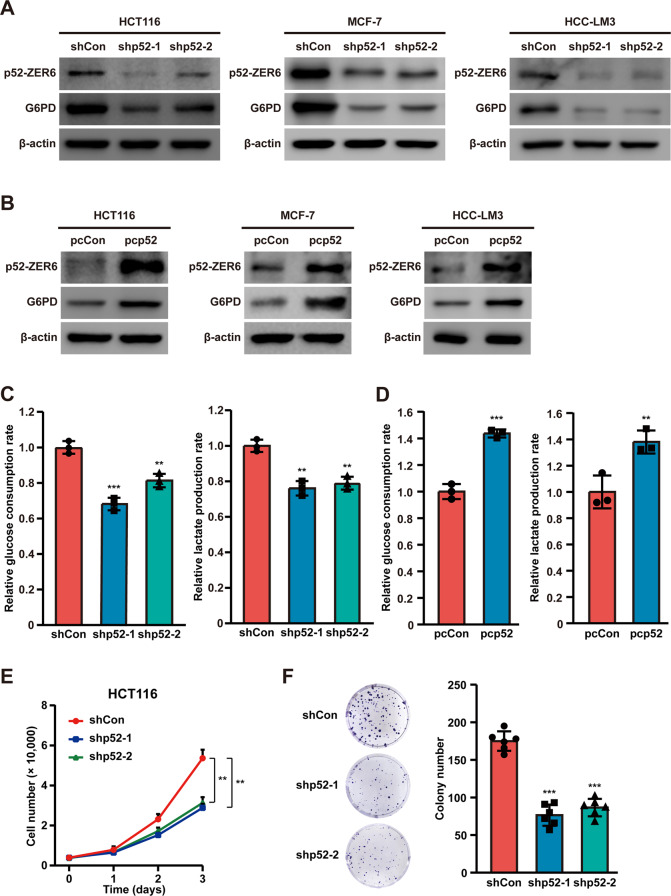
p52-ZER6 positively regulates G6PD expression and enhances tumor cells glucose metabolism. **A** G6PD protein expression levels in *p52*-*ZER6*-silenced HCT116, MCF-7, and HCC-LM3 cells, as determined using western blotting. **B** G6PD protein expression levels in *p52*-*ZER6*-overexpressed HCT116, MCF-7, and HCC-LM3 cells, as determined using western blotting. **C**, **D** Glucose consumption (left) and lactate production (right) levels in *p52-ZER6*-silenced (**C**) and *p52-ZER6*-overexpressed (**D**) HCT116 cells. **E** Viability of *p52-ZER6*-silenced HCT116 cells at indicated time points. **F** Colony formation potential of *p52-ZER6*-silenced HCT116 cells. Representative images (left) and quantification results (right; *n* = 6) are shown. Cells transfected with shCon or pcCon were used as controls. β-actin was used as western blotting loading control. Total protein was used for normalizing the levels of glucose consumption and lactate production. Quantification data are expressed as mean ± SD (*n* = 3, unless otherwise indicated). shp52: shRNA expression vector targeting *p52-ZER6*; pcCon: pcEF9-Puro; pcp52: *p52-ZER6* overexpression vector; **P < 0.01; ***P < 0.001.

We next investigated whether p52-ZER6 regulation of G6PD was limited to colorectal tumor cells or was found in various cancers. As shown in Fig. 2A, knocking down *p52-ZER6* clearly suppressed the expression of G6PD in breast cancer and hepatocellular cancer cells, while its overexpression had the opposite effect (Fig. 2B and Fig. S3A), suggesting that such regulatory action was common to various cancer types. Knocking down *p52-ZER6* clearly suppressed glucose consumption and lactate production (Fig. 2C); whereas overexpressing this gene conspicuously increased these rates (Fig. 2D). Furthermore, altered *p52-ZER6* expression correlated positively with G6PD enzymatic activity in HCT116, MCF-7, and HCC-LM3 cells (Figs. S3B–S3D). Subsequently, we showed that knocking down *p52-ZER6* suppressed HCT116 cell viability and colony formation potential (Figs. 2E, 2F); while inducing cell cycle arrest (Fig. S3E). Meanwhile, although knocking down *p71-ZER6* could suppress cell viability (Fig. S4A), it failed to affect G6PD expression level (Figs. 1J, 1K) as well as its enzymatic activity (Fig. S4B), again confirming that the regulatory effect on G6PD was specific to p52-ZER6. In summary, these results indicate that p52-ZER6 enhances G6PD expression and positively contributes to tumorigenic potential.

### G6PD is crucial for p52-ZER6-mediated PPP activation

**Fig. 3:**
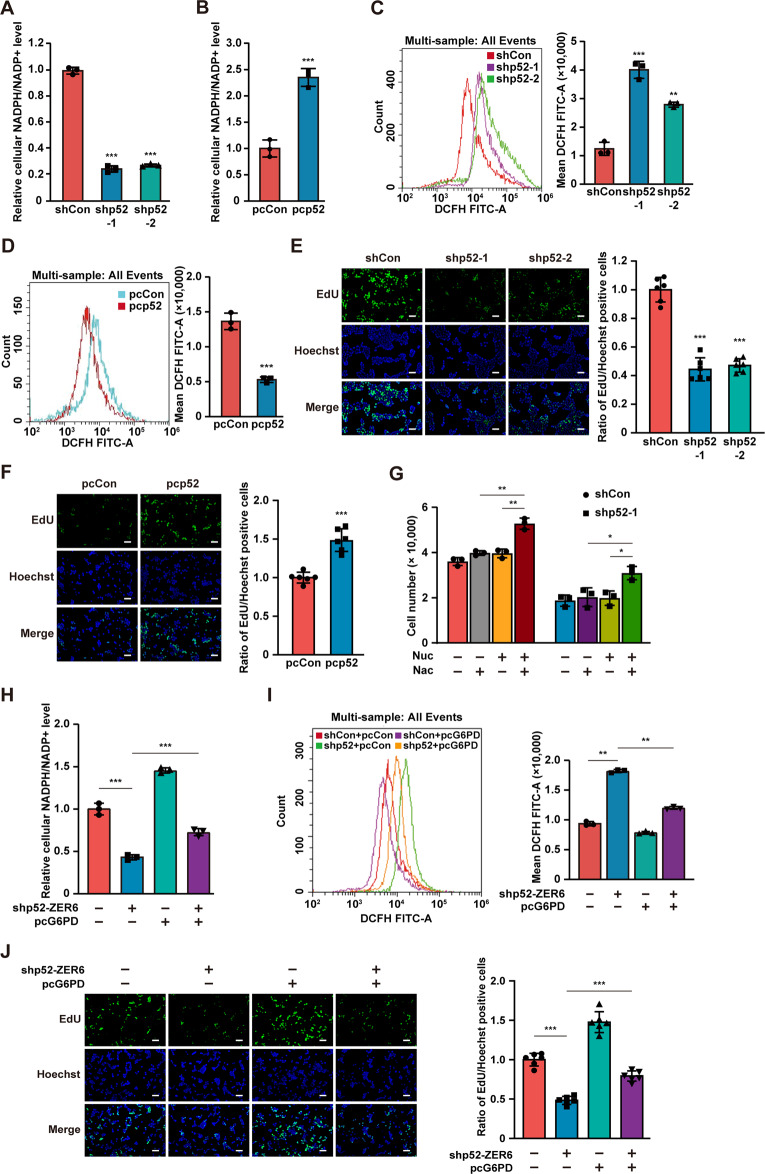
G6PD is crucial for p52-ZER6 regulation on PPP. **A**, **B** Intracellular NADPH/NADP^+^ ratio in *p52-ZER6*-silenced (**A**) and *p52-ZER6*-overexpressed (**B**) HCT116 cells. **C**, **D** Intracellular ROS levels in *p52-ZER6*-silenced (**C**) and *p52-ZER6*-overexpressed (**D**) HCT116 cells. **E**, **F** DNA replication in *p52-ZER6*-silenced (**E**) and *p52-ZER6*-overexpressed (**F**) HCT116 cells, as determined using EdU incorporation assay. Representative images (left; scale bars: 50 μm) and quantitative results (right; *n* = 6) are shown. **G** Viability of *p52-ZER6*-silenced HCT116 cells cultured in the presence of nucleosides mixture (Nuc) and/or ROS scavenger (N-acetyl-L-cysteine, Nac), as measured at the third day after the addition of nucleosides mixture and/or ROS scavenger. **H**, **I** Intracellular NADPH/NADP^+^ ratio (**H**) and ROS level (**I**) in *p52-ZER6*-silenced, *G6PD*-overexpressed HCT116 cells. **J** DNA replication in *p52-ZER6*-silenced, *G6PD*-overexpressed HCT116 cells, as determined using EdU incorporation assay. Representative images (left, scale bars: 50 μm) and quantitative results (right, *n* = 6) are shown. Cells transfected with shCon and/or pcCon were used as controls. Quantification data are expressed as mean ± SD (*n* = 3, unless otherwise indicated). shp52: shRNA expression vector targeting *p52-ZER6*; pcCon: pcEF9-Puro; pcp52: *p52-ZER6* overexpression vector; *P < 0.05; **P < 0.01; ***P < 0.001.

As the first rate-limiting enzyme in the PPP, G6PD plays an important role in glycolytic metabolism. Given that p52-ZER6 positively regulates G6PD levels, we investigated the effect of p52-ZER6 on NADPH, a metabolic intermediate of the PPP that protects tumor cells from oxidative stress. As shown in Figs. S5A and S5B, *p52-ZER6* silencing significantly reduced cellular NADPH levels while increasing NADP^+^ levels. In contrast, *p52-ZER6* overexpression increased cellular NADPH levels (Fig. S5C) and suppressed NADP^+^ levels (Fig. S5D). This resulted in a decrease in the NADPH/NADP^+^ ratio in *p52-ZER6*-silenced HCT116 cells (Fig. 3A) and an increase in this ratio in *p52-ZER6*-overexpressing cells (Fig. 3B). Consequently, while *p52-ZER6* silencing led to an increase in cellular ROS, *p52-ZER6* overexpression suppressed it (Figs. 3C, 3D).

In addition to NADPH, ribose-5-phosphate, which plays a crucial role by providing the building blocks for DNA synthesis, is an important intermediate of the PPP pathway. In contrast to the effect seen when knocking down *p52-ZER6* (Fig. 3E), its overexpression robustly increased the ratio of 5-ethynyl-2′-deoxyuridine (EdU)-positive cells (Fig. 3F). This suggests that p52-ZER6 has a positive regulatory effect on DNA replication.

To examine the regulatory role of p52-ZER6 in the PPP with respect to its oncogenic function, we performed rescue experiments using N-acetyl-L-cysteine, a ROS scavenger, and a mixture of four ribonucleosides and four deoxyribonucleosides to counteract the decrease in nucleic acid biosynthesis. The addition of both N-acetyl-L-cysteine and the nucleoside mixture significantly reversed the effect of knocking down *p52-ZER6* on HCT116 cells (Fig. 3G). Of note, the addition of either N-acetyl-L-cysteine or the nucleoside mixture alone failed to exert a significant effect, suggesting that both the suppression of ROS and sufficient nucleic acid biosynthesis are indispensable to restore the viability of *p52-ZER6*-silenced tumor cells. Together, these results suggest that p52-ZER6 positively regulates the PPP in tumor cells, which is crucial in promoting tumor cell proliferation and viability.

Next, to determine whether G6PD was involved in p52-ZER6-mediated PPP regulation, we performed rescue experiments by transfecting both *p52-ZER6*-silencing and *G6PD*-overexpression vectors into HCT116 cells (Fig. S6A) and confirmed the restoration of G6PD activity (Fig. S6B). *G6PD* overexpression led to an increase in cellular NADPH and a decrease in NADP^+^ that were altered by *p52-ZER6* silencing (Figs. S6C and S6D), thereby enhancing the NADPH/NADP^+^ ratio (Fig. 3H). In line with this observation, *G6PD* overexpression suppressed cellular ROS levels induced by *p52-ZER6* silencing (Fig. 3I). Similarly, G6PD restored the ratio of EdU-positive cells, which was suppressed by *p52-ZER6* silencing (Fig. 3J). Furthermore, *G6PD* overexpression restored cell viability and colony formation potential in *p52-ZER6*-silenced HCT116 cells (Fig. S7A and S7B). Collectively, these results show that p52-ZER6-mediated regulation of G6PD is crucial for promoting PPP activity and tumorigenesis.

### p52-ZER6 regulates G6PD and the PPP in a p53-independent manner

**Fig. 4:**
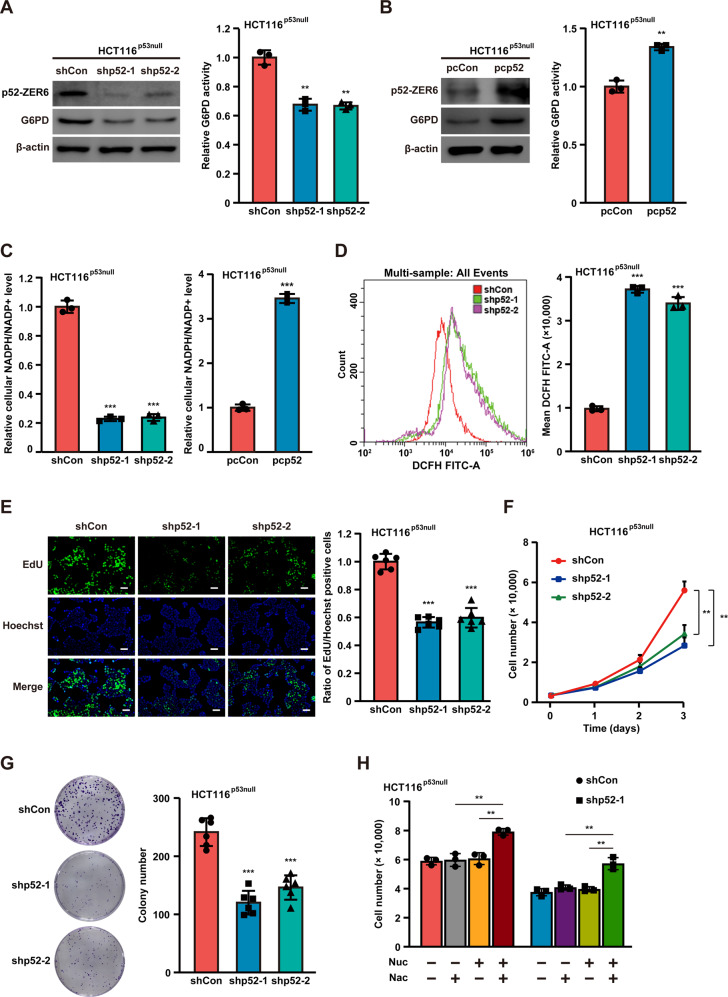
p52-ZER6 regulation on G6PD/PPP occurs in a p53-independent manner. **A**, **B** G6PD protein (left) and enzymatic activity (right) in *p52-ZER6*-silenced (**A**) and *p52-ZER6*-overexpressed (**B**) HCT116^p53null^ cells. **C** Intracellular NADPH/NADP^+^ ratio in *p52-ZER6*-silenced (left) and *p52-ZER6*- overexpressed (right) HCT116^p53null^ cells. **D** Intracellular ROS level in *p52-ZER6*-silenced HCT116^p53null^ cells. **E** DNA replication in *p52-ZER6*-silenced HCT116^p53null^ cells, as determined using EdU incorporation assay. Representative images (left, scale bars: 50 μm) and quantitative results (right; *n* = 6) are shown. **F** Viability of *p52-ZER6*-silenced HCT116^p53null^ cells at indicated time points. **G** Colony formation potential of *p52-ZER6*-silenced HCT116^p53null^ cells. Representative images (left) and quantification results (right, *n* = 6) are shown. **H** Viability of *p52-ZER6*-silenced HCT116^p53null^ cells cultured in the presence of nucleosides mixture (Nuc) and/or ROS scavenger (N-acetyl-L-cysteine, Nac), as measured at the third day after the addition of nucleosides mixture and/or ROS scavenger. Cells transfected with shCon or pcCon were used as controls. β-actin was used as western blotting loading control. Total protein was used for normalizing the level of G6PD enzymatic activity. Quantification data are expressed as mean ± SD (*n* = 3, unless otherwise indicated). shp52: shRNA expression vector targeting *p52-ZER6*; pcCon: pcEF9-Puro; pcp52: *p52-ZER6* overexpression vector; **P < 0.01; ***P < 0.001.

The tumor suppressor p53 inhibits the PPP by blocking G6PD expression [16]. Our previous study revealed that p52-ZER6 could enhance p53 protein ubiquitination and destabilize it by strengthening the binding of p53 to its E3 ligase, mouse double minute 2 (MDM2) [12]. Here, we examined whether the p52-ZER6-mediated regulation of G6PD occurred in a p53-dependent manner. To this end, we knocked down *p52-ZER6* in p53-null HCT116 (HCT116^p53null^) cells (Figs. S8A, S8B). Surprisingly, we found that *p52-ZER6* silencing significantly suppressed *G6PD* mRNA expression in the absence of p53 (Fig. S8C). Similarly, *p52-ZER6* silencing robustly suppressed G6PD protein expression and enzymatic activity in HCT116^p53null^ cells (Fig. 4A). In agreement with this finding, *p52-ZER6* overexpression elevated G6PD levels and enzymatic activity in HCT116^p53null^ cells (Fig. S8D and Fig. 4B). Together, these results reveal that p52-ZER6 regulates G6PD expression in a p53-independent manner.

Next, we investigated whether p52-ZER6 could regulate the PPP via a p53‐independent pathway. Even in the absence of p53, *p52-ZER6* silencing significantly decreased cellular NADPH levels and increased NADP^+^ levels (Fig. S8E); whereas *p52-ZER6* overexpression achieved the opposite outcome (Fig. S8F). Accordingly, the NADPH/NADP^+^ ratio was decreased in *p52-ZER6*-silenced HCT116^p53null^ cells and increased in *p52-ZER6-*overexpressing HCT116^p53null^ cells (Fig. 4C). Concomitantly, *p52-ZER6* silencing increased intracellular ROS levels in HCT116^p53null^ cells (Fig. 4D). The EdU incorporation assay showed that *p52-ZER6* silencing could significantly suppress DNA replication in HCT116^p53null^ cells (Fig. 4E). A similar trend was observed with respect to cell viability and colony formation potential (Figs. 4F and 4G); whereas the addition of N-acetyl-L-cysteine and nucleosides restored the viability of HCT116^p53null^ cells (Fig. 4H). Together, these results strongly indicate that p52-ZER6 regulates G6PD and activates the PPP in a p53-independent manner.

### p52-ZER6 promotes *G6PD* transcriptional activity

**Fig. 5:**
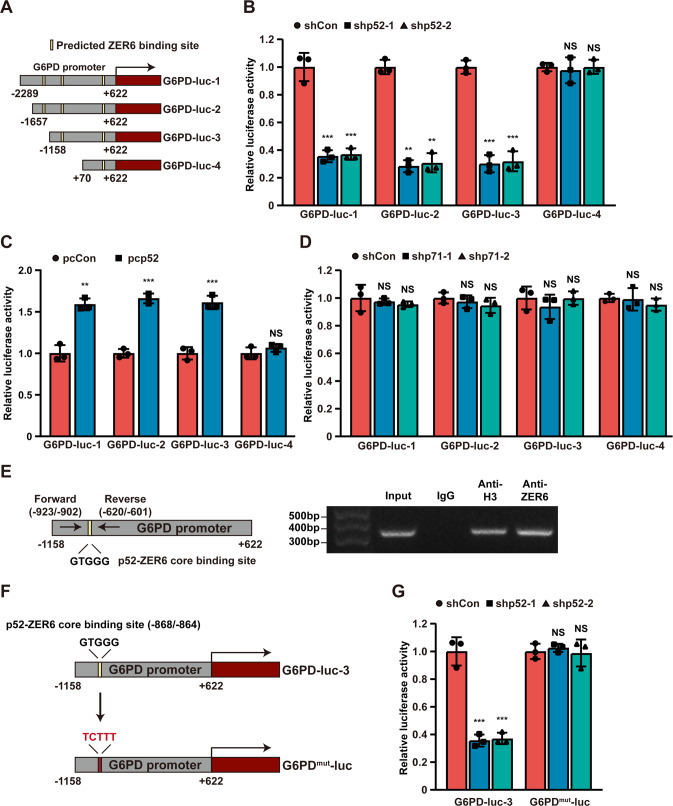
p52-ZER6 directly promotes *G6PD* transcriptional activity by binding to its promoter. **A** Schematic diagram of the luciferase reporter vectors carrying various *G6PD* promoter regions. **B**, **C** Relative luciferase activities of G6PD-luc-1 to G6PD-luc-4 in *p52-ZER6*-silenced (**B**) and *p52-ZER6*-overexpressed (**C**) HCT116 cells. **D** Relative luciferase activities of G6PD-luc-1 to G6PD-luc-4 in *p71-ZER6*-silenced HCT116 cells. **E** Binding of p52-ZER6 to the promoter region of *G6PD*, as examined using ChIP assay with anti-ZER6 antibody and followed by PCR. The predicted p52-ZER6 binding site on the promoter region of *G6PD* and the location of primer set used for PCR are shown. Anti-histone H3 antibody was used as a positive control. **F**, **G** Relative luciferase activities of G6PD^mut^-luc in *p52-ZER6*-silenced HCT116 cells. Schematic diagram (**F**) and luciferase activities (**G**) are shown. Mutated base pairs are indicated in red. Cells transfected with shCon or pcCon were used as controls. Luciferase activities are shown as relative to control. Quantification data are expressed as mean ± SD (*n* = 3). shp52: shRNA expression vector targeting *p52-ZER6*; shp71: shRNA expression vector targeting *p71-ZER6*; pcCon: pcEF9-Puro; pcp52: *p52-ZER6* overexpression vector; **P < 0.01; ***P < 0.001; NS not significant.

To further explore the molecular mechanism by which p52-ZER6 regulates G6PD, we predicted four ZER6-binding elements in the *G6PD* promoter using a eukaryotic promoter database (www.epd.isb-sib.ch/) [11]. Accordingly, we constructed four luciferase reporter assay vectors carrying different regions of the *G6PD* promoter: G6PD-luc-1, which contained the -2,289 to +622 region; G6PD-luc-2, which contained the −1657 to +622 region; G6PD-luc-3, which contained the −1158 to +622 region; and G6PD-luc-4, which contained the +70 to +622 region (Fig. 5A). Luciferase assays revealed that *p52-ZER6* silencing significantly suppressed the activity of G6PD-luc-1, G6PD-luc-2, and G6PD-luc-3, but not that of G6PD-luc-4 (Fig. 5B). Similarly, *p52-ZER6* overexpression significantly promoted G6PD-luc-1, G6PD-luc-2, and G6PD-luc-3 activities, but not that of G6PD-luc-4 (Fig. 5C), suggesting that the −1158 to +69 position of the *G6PD* promoter was crucial for its transcriptional regulation by p52-ZER6. Notably, *p71-ZER6* silencing failed to exert a significant effect on all G6PD-luc reporters (Fig. 5D), further confirming that the p52-ZER6-specific regulatory effect of ZER6 on *G6PD*.

To determine whether p52-ZER6 could bind directly to the putative p52-ZER6-binding site in the *G6PD* promoter region, we performed a chromatin immunoprecipitation (ChIP) assay and found that G6PD bound to the −923 to −601 region of the *G6PD* promoter (Fig. 5E). Subsequently, we constructed a G6PD luciferase reporter vector with five mutations in the p52-ZER6 core binding site (G6PD^mut^-luc), whereby the GTGGG core motif at positions −868 to −864 was mutated to TCTTT (Fig. 5F). The luciferase assay revealed that whereas knocking down *p52-ZER6* significantly suppressed the activity of G6PD-luc-3, it failed to alter the activity of G6PD^mut^-luc (Fig. 5G). Together, these results indicate that p52-ZER6 could promote G6PD expression by binding directly to the *G6PD* promoter and that such binding is critical for transcriptional regulation.

### The p52-ZER6/G6PD axis enhances tumorigenesis in a p53-independent manner

**Fig. 6:**
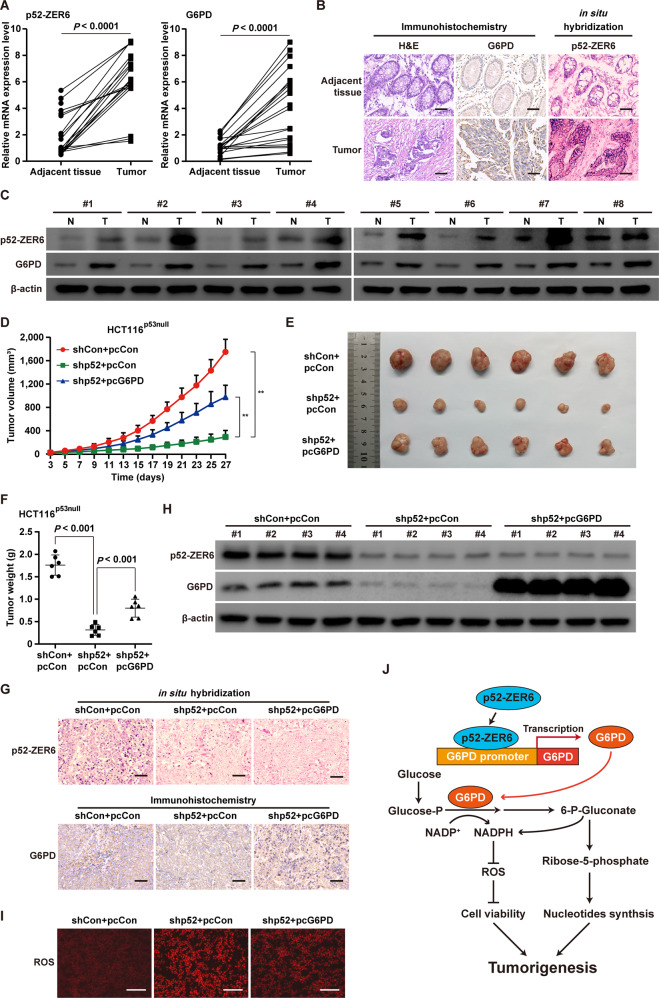
p52-ZER6/G6PD axis enhances the tumorigenic potential of CRC cells in a p53-independent manner. **A** p52-ZER6 and G6PD mRNA expression levels in clinical colorectal cancer and corresponding adjacent tissues, as analyzed using qRT-PCR (*n* = 21). **B** Expression levels of p52-ZER6 and G6PD in clinical colorectal cancer and corresponding adjacent tissue, as examined using in situ hybridization assay and immunohistochemistry staining using serial sections, respectively (scale bars: 50 μm). **C** p52-ZER6 and G6PD protein expression levels in clinical colorectal cancer and corresponding adjacent tissues, as determined using western blotting (*n* = 8). **D**, **F** Tumorigenesis potentials of *p52*-*ZER6*-knocked down HCT116^p53null^ and *p52-ZER6*-knocked down, *G6PD*-overexpressed HCT116^p53null^ stable cell lines, as examined in vivo by xenograft experiments (n = 6). Tumor volume at indicated time points (**D**), representative morphological images (**E**), and tumor weight (**F**) at day 27 after transplantation are shown. **G** Expression levels of p52-ZER6 and G6PD in the xenografted tumors formed by indicated cells, as examined using in situ hybridization assay and immunohistochemistry staining using serial sections, respectively (scale bars: 50 μm). **H** p52-ZER6 and G6PD protein expression levels in the xenografted tumors formed by indicated cells, as determined using western blotting. **I** ROS levels in the xenografted tumors formed by indicated cells (scale bars: 100 μm). **J** Schematic diagram showing the molecular mechanism of p52-ZER6 on CRC tumorigenesis via direct transcriptional regulation on *G6PD*. Cells transfected with shCon and pcCon were used as controls. β-actin was used for qRT-PCR normalization and as western blotting loading control. Quantification data are expressed as mean ± SD. shp52: shRNA expression vector targeting *p52-ZER6*; pcCon: pcEF9-Puro; pcG6PD: *G6PD* overexpression vector; **P < 0.01.

To determine the role of the p52-ZER6/G6PD axis in tumorigenesis, we first analyzed the mRNA levels of *p52-ZER6* and *G6PD* in clinical colorectal cancer tissues. Expression of both *p52-ZER6* and *G6PD* was significantly upregulated in colorectal cancer tissues compared to adjacent non-cancerous tissues (Fig. 6A). This trend was confirmed by in situ hybridization and immunohistochemistry staining using serial sections obtained from clinical colorectal cancer tissue and the corresponding normal adjacent tissue (Fig. 6B), and by western blotting (Fig. 6C). Therefore, these results point to a positive correlation between p52-ZER6 and G6PD expression in clinical colorectal cancer patient samples.

Finally, we performed xenograft experiments to examine the role of the p52-ZER6/G6PD axis in tumorigenesis in vivo. For this purpose, we established *p52-ZER6*-silenced, *G6PD*-overexpressing HCT116^p53null^ stable cell lines (Fig. S8). Subcutaneous transplantation of these cells into BALB/c-nu/nu mice revealed that *p52-ZER6* silencing decreased the volume of the tumors formed, whereas *G6PD* overexpression largely restored it (Fig. 6D), suggesting that knocking down *p52-ZER6* could slowdown tumor growth, most plausibly by suppressing the PPP. This result was supported by assessing tumor morphology and weight (Figs. 6E and 6F). Using serial sections of the xenografted tumor, we performed in situ hybridization and immunohistochemistry staining, and confirmed a decrease in p52-ZER6 and G6PD expression in xenografted tumors formed by *p52-ZER6*-silenced HCT116^p53null^ cells (Fig. 6G). Concomitantly, a decrease in G6PD protein levels was observed by western blotting (Fig. 6H). Finally, *G6PD* overexpression suppressed ROS production induced by *p52-ZER6* silencing (Fig. 6I).

In summary, our findings reveal a novel function of p52-ZER6 in inducing tumor cell metabolic reprogramming by directly promoting *G6PD* transcription. This regulatory event induces the PPP, which provides reductants to protect cells from ROS damage, as well as building blocks for nucleic acid precursors, ultimately promoting tumorigenesis (Fig. 6J).

## Discussion

Cancer results from cell cycle disorders and unlimited cell proliferation [17]. To ensure their survival, tumor cells must satisfy their bioenergy requirements and mitigate oxidative stress [18]. Glucose metabolic reprogramming is an important feature of tumor cells by which they meet their energy requirements [19] and obtain building blocks for the biosynthesis of macromolecules required to support their rapid proliferation [20]. Moreover, glucose metabolic reprogramming is also closely related to tumor cell drug resistance [21] and has a profound stimulatory effect on tumor progression, as changes in intracellular and extracellular metabolites can directly or indirectly affect oncogenic mutations and enhance nutrient absorption, thereby further providing energy and precursors [22–24]. Despite its importance, the regulation of glucose metabolic reprogramming in tumor cells has not been completely elucidated. Previous studies have revealed that C_2_H_2_ zinc finger proteins such as zinc finger E-box-binding homeobox 1 (ZEB1), ZNF475 and ZNF568 could enhance tumor cell glycolysis [13, 14]. The present study shows that p52-ZER6 is crucial for tumor metabolic reprogramming, especially via the PPP, as indicated by upregulation of glucose consumption and lactate production in tumor cells. Furthermore, we show that this regulation might be a general mechanism in various tumors. Given that p52-ZER6 expression is upregulated in breast cancer [25] and colon cancer [12], our findings suggest a crucial role for this axis in altering glucose metabolism in tumor cells. Importantly, although less significant than its effect on G6PD, p52-ZER6 might also affect the expression of other glucose metabolism-related genes, such as *FH*, *PKM2*, *TIGAR*, *LDHA*, *GLUT1*, and *HK2*, thereby contributing to tumor metabolic reprogramming through multiple pathways.

The regulatory effect on G6PD is specific for p52-ZER6, as p71-ZER6 failed to regulate G6PD expression or its activity. While this and a previous study show that p71-ZER6 could enhance tumor cell proliferation and colony formation [12], it is likely that p71-ZER6 is involved in tumorigenesis through a pathway other than G6PD/PPP.

The PPP as a hexose monophosphate shunt of the glycolysis pathway, is initiated by the G6PD-mediated conversion of glucose-6-phosphate, an intermediate of glycolysis, to 6-phospho-gluconolactone. The PPP can provide tumor cells with the cellular reductants NADPH and ribose-5-phosphate. NADPH is required for fatty acid synthesis and ROS scavenging [7, 26]. Meanwhile, ribose-5-phosphate provides the building blocks for nucleic acid biosynthesis. Hence, the PPP allows tumor cells to defend themselves against increased ROS levels caused by a rapid metabolism, as well as meet the elevated demand for nucleic acid and fatty acid biosynthesis [6, 7, 27]. Furthermore, the PPP has attracted attention as a potential target for antitumor therapy [28]. Our study indicates that G6PD, the first rate-limiting enzyme of the PPP, is a target of p52-ZER6, which binds to the *G6PD* promoter and enhances its transcriptional activity. Indeed, increasing the PPP flux via G6PD stimulation has been found to trigger cancer progression in a variety of tumor types [29–31], coordinate tumor cell cycle progression [32, 33], promote antioxidant defense and metastasis [8, 34], and increase drug resistance [35, 36]. Meanwhile, inhibition of G6PD can lead to accumulation of reactive oxygen species, induce endoplasmic reticulum stress, and slow the growth and migration of cancer cells [9, 37]. G6PD ubiquitination inhibits the PPP pathway and the malignant phenotype of tumors [38], reflecting its importance in PPP regulation and subsequent tumorigenesis. Interestingly, we show here that the p52-ZER6-mediated regulation of G6PD, and subsequently the PPP, is independent of p53. Given that p53 can bind to G6PD and prevent the formation of an active dimer [16], while p52-ZER6 can enhance p53 protein ubiquitination/proteasomal degradation by strengthening its binding to MDM2 [39], our results suggest the presence of both p53-dependent and p53-independent pathways in the p52-ZER6-mediated regulation of G6PD. Hence, whereas further preclinical and clinical studies are necessary, our study suggests a potential antitumor therapeutic strategy based on targeting the p52-ZER6/G6PD/PPP axis both in patients with wild-type *p53* and in those with mutant *p53*. This is of particular clinical importance as more than 50% of tumor patients harbor mutations in the *p53* gene and wild-type *p53* expression is aberrantly low, suggesting abnormalities in the *p53* regulatory mechanism [39, 40]. Furthermore, in this study, we showed that p52-ZER6 could enhance *G6PD* expression in estrogen receptor (ER)-positive breast cancer cells MCF-7. Given that previous studies have reported both estradiol-dependent and estrogen receptor-independent regulation of G6PD expression [41, 42], and that p52-ZER6 could bind to *G6PD* promoter and induce its activity, these facts point out the possibility of the presence of both estrogen/estrogen receptor-dependent and -independent regulation of G6PD.

In conclusion, we report an unprecedented link between p52-ZER6 and tumor cell metabolic reprogramming, which involves activation of the G6PD/PPP axis and subsequent tumorigenesis. The present findings provide new insights into the regulation of G6PD/PPP, as well as the biological and pathological functions of p52-ZER6. Furthermore, we suggest the potential of targeting *p52-ZER6* for the diagnosis and treatment of tumors, as well as metabolic disorders.

## Supplementary information

Supplementary Materials

## Data Availability

The datasets used in this analysis are available from the corresponding authors upon reasonable request.
