# Supplementary material for: The p52-ZER6/G6PD axis alters aerobic glycolysis and promotes tumor progression by activating the pentose phosphate pathway
Source: Oncogenesis. 2023 Mar 28;12(1):17. doi: 10.1038/s41389-023-00464-4 (PMC10050210; doi:10.1038/s41389-023-00464-4)
Supplement: Supplementary file 1 — Supplementary Materials [file 41389_2023_464_MOESM1_ESM.pdf]

Supplementary Materials for

p52-ZER6/G6PD axis alters aerobic glycolysis and promotes tumor progression by activating pentose phosphate pathway

Yu Tang, Wenfang Li, Li Qiu, Xia Zhang, Lei Zhang,
Makoto Miyagishi, Hezhao Zhao, Shourong Wu*, Vivi Kasim*

*Email: shourongwu@cqu.edu.cn
vivikasim@cqu.edu.cn

This PDF file includes:

Supplementary Materials and Methods

Figure S1. The efficacy of shRNA expression vectors targeting *ZER6* in HCT116 cells.

Figure S2. The efficacy of shRNA expression vectors targeting p52-ZER6 and p71-ZER6 in HCT116 cells.

Figure S3. p52-ZER6 promotes G6PD expression and enzymatic activity.

Figure S4. p71-ZER6 fails to promote G6PD enzymatic activity.

Figure S5. Effect of p52-ZER6 alteration on intracellular NADPH and $NADP^+$ levels.

Figure S6. G6PD is crucial for p52-ZER6 regulation on cellular NADPH and $NADP^+$ levels.

Figure S7. G6PD is crucial for p52-ZER6 oncogenic potential.

Figure S8. p52-ZER6 regulates G6PD and PPP in a p53-independent manner.

Figure S9. Establishment of *p52-ZER6*-silenced, *G6PD*-overexpressed HCT116^{p53null} stable cell line.

Table S1. Primer pairs used for qRT-PCR.

Table S2. Antibodies used for western blotting, ChIP assay, immunohistochemistry and *in situ* hybridization assay.

Supplementary Materials and Methods

ChIP assay

ChIP analysis was performed using the ChIP-IT Express (Beyotime Biotechnology) according to the manufacturer's instructions. Briefly, to crosslink proteins to DNA, formaldehyde (final concentration 1%) was added to the culture medium of HCT116 cells overexpressing *p52-ZER6*. Cells were then collected, and the pellets were treated with Lysis Buffer prior to sonication using ultrasonic crusher (Chongqing Medical Equipment Factory, Chongqing, China) to shear DNA into 0.2-1.0 kb fragments. Sonication was performed for 10 s (power: 150 W) followed by 10 s interval on ice, and was repeated 10 times. After the cellular debris was removed, the chromatins were immunoprecipitated using protein A+G Agarose/Salmon Sperm DNA (Beyotime Biotechnology) and anti-ZER6 antibody, anti-Histone H3 antibody or normal rabbit IgG. Chromatin was then de-crosslinked for 4 h at 65°C, and treated with 5 M NaCl, 0.5 M EDTA, 1 M Tris (pH 6.5), and 20 mg/ml proteinase K. Immunoprecipitated chromatin was then subjected to PCR analysis using PrimeSTAR Max (Takara Bio). The sequence of the forward primer used was 5'-TGT CTT TGG GGA AAA GGA CCA C-3'; while that of the reverse primer was 5'- GGC CGG CGT GCT TAT CAT TA -3'.

RNA extraction and quantitative real time PCR (qRT-PCR) analysis

Total RNA was extracted with Trizol (Invitrogen Life Technologies) according to the manufacturer's instruction. Total RNA (1 μg) was reverse-transcribed into cDNA using the PrimeScript RT Reagent Kit with gDNA Eraser (Takara Bio), and qRT-PCR was performed with SYBR Premix Ex Taq (Takara Bio). The sequences of the primers used for qRT-PCR were

shown in Supplementary Table S1. β-actin was used to normalize sample amplifications. The results are shown as relative to the expression level in the corresponding controls, which were assumed as 1.

Western blotting

For cell culture experiments, cells were collected and lysed with RIPA lysis buffer with protease inhibitor and phosphatase inhibitor cocktail (complete cocktail; Roche Applied Science, Mannheim, Germany). For clinical specimens and samples from xenografted tumors, frozen specimens were homogenized with RIPA lysis buffer with protease inhibitor and phosphatase inhibitor cocktail to obtain protein extracts. Equal amounts of total protein (20 μg) were electrophoresed on sodium dodecyl sulfate polyacrylamide gel and transferred to a polyvinylidene fluoride (PVDF) membrane (Millipore, Billerica, MA). The antibodies used are listed in Supplementary Table S2 and immunoblotting with anti-β-actin antibody was conducted to ensure equal protein loading. The signals were detected by using SuperSignal West Femto Maximum Sensitivity Substrate detection system (Thermo Scientific, Waltham, MA). Quantification was performed using Quantity One, and the result was normalized using β-actin.

Immunohistochemistry

Tissue sections were obtained from fresh colon carcinoma and adjacent tissues or xenografted tumors at 4 μm thickness using a cryostat and subjected to immunohistochemistry. Briefly, the tissue sections were incubated with primary antibodies for 1 h. The specimens were then

incubated with corresponding second antibodies conjugated with horse-radish peroxidase. Visualization was performed using a DAB Kit (DAKO, Denmark) under microscope. The nuclei were then counterstained with hematoxylin, followed by dehydration and coverslip mounting. The antibodies used were listed in Table S2. Images were taken by using Pannoramic Midi (3DHistech).

Glucose consumption, lactate production, G6PD enzyme activity, and intracellular NADPH level

Cells were transfected with indicated vectors and selected using puromycin as indicated above. For measuring glucose consumption and lactate production, the medium was replaced after selection, and the cells were incubated for an additional 24 h. Glucose and lactate levels in the culture medium were determined using the Glucose Colorimetric Assay Kit (BioVision, Milpitas, USA) and Lactate Assay Kit (KeyGen Biotech, Jiangsu, China), respectively, according to the manufacturers' instructions. G6PD enzyme activity and intracellular NADPH level were determined using G6PD Assay Kit (Yuanye, Shanghai, China) and Amplite^{TM} Colorimetric NADPH/NADP^{+} Ratio Assay Kit (Comin Bio, Suzhou, China), respectively, according to the manufacturers' instructions. The values were normalized with total protein amount determined using the BCA Protein Assay Kit (Beyotime Biotechnology).

5-ethynyl-2′-deoxyuridine (EdU) incorporation assay

Cells were transfected with indicated vectors and selected using puromycin as indicated above prior being re-seeded in 24-well plate (1×10^5 cells/well). EdU incorporation assay was

performed using BeyoClick^{TM} EdU Cell Proliferation Kit with Alexa Flour 488 (Beyotime Biotechnology) according to the manufacturer's instruction. Hoechst was used to stain the nuclei. Images were taken with DMI6000B (Leica, Heidelberg, Germany). The ratio of *de novo* DNA synthesis was calculated as the ratio of EdU-positive cells to Hoechst-positive cells.

Dual luciferase reporter assay

Cells were co-transfected with indicated vectors, reporter vector bringing the firefly luciferase, and *Renilla* luciferase expression vector pRL-SV40 (Promega). After 24 h, luciferase activities were analyzed by using Dual Luciferase Reporter Assay (Promega). The activities of the firefly luciferase reporters were normalized using those of *Renilla* luciferase.

Colony formation assay

Cells were transfected with indicated shRNA expression vectors or overexpression vector and screened using puromycin as indicated above. After being re-seeded into 6-well plates at a density of 300 cells/well and cultured for 12 days, cells were then fixed with 4% paraformaldehyde and stained with Crystal Violet Staining Solution (Beyotime Biotechnology), then the numbers of the colonies formed were counted. The investigator was blinded during the assessment.

Cell viability

Cells were transfected with indicated shRNA expression vectors or overexpression vector and selected using puromycin as indicated above. Cells were re-seeded in 96-well plates (3×10^3

cells/well) and counted at indicated time points using colorimetric assay with 3-(4,5-dimethylthiazol-2-yl)-5-(3-carboxymethoxyphenyl)-2-(4-sulfophenyl)-2H-tetrazolium (MTS, Promega) in accordance with the manufacturer's instructions. For experiment with nucleosides, cells were re-seeded in 96-well plates at a density of 3×10^3 cells/well and cultured with medium containing 200 μM (final concentration) mixture of 4 ribonucleosides (adenosine, uridine, cytidine, guanosine; Sigma Aldrich) and 4 deoxyribonucleosides (deoxyadenosine, deoxyguanosine, deoxythymidine, and deoxycytidine; Sigma Aldrich) in the presence or absence of ROS scavenger N-acetyl-L-cysteine (final concentration: 2 mM; Sigma Aldrich). Cell numbers were counted as described above three days after being re-seeded.

Cell cycle analysis

Cells were transfected with indicated shRNA expression vectors or overexpression vector. Transfected cells were selected using puromycin as described above and subjected to starvation for 24 h before being incubated further for 24 h in medium containing 10% FBS. Cells were then harvested and stained with propidium iodide (Solarbio, Beijing, China). The percentages of the cells in each cell cycle phase were determined by flow cytometry.

Figure S1

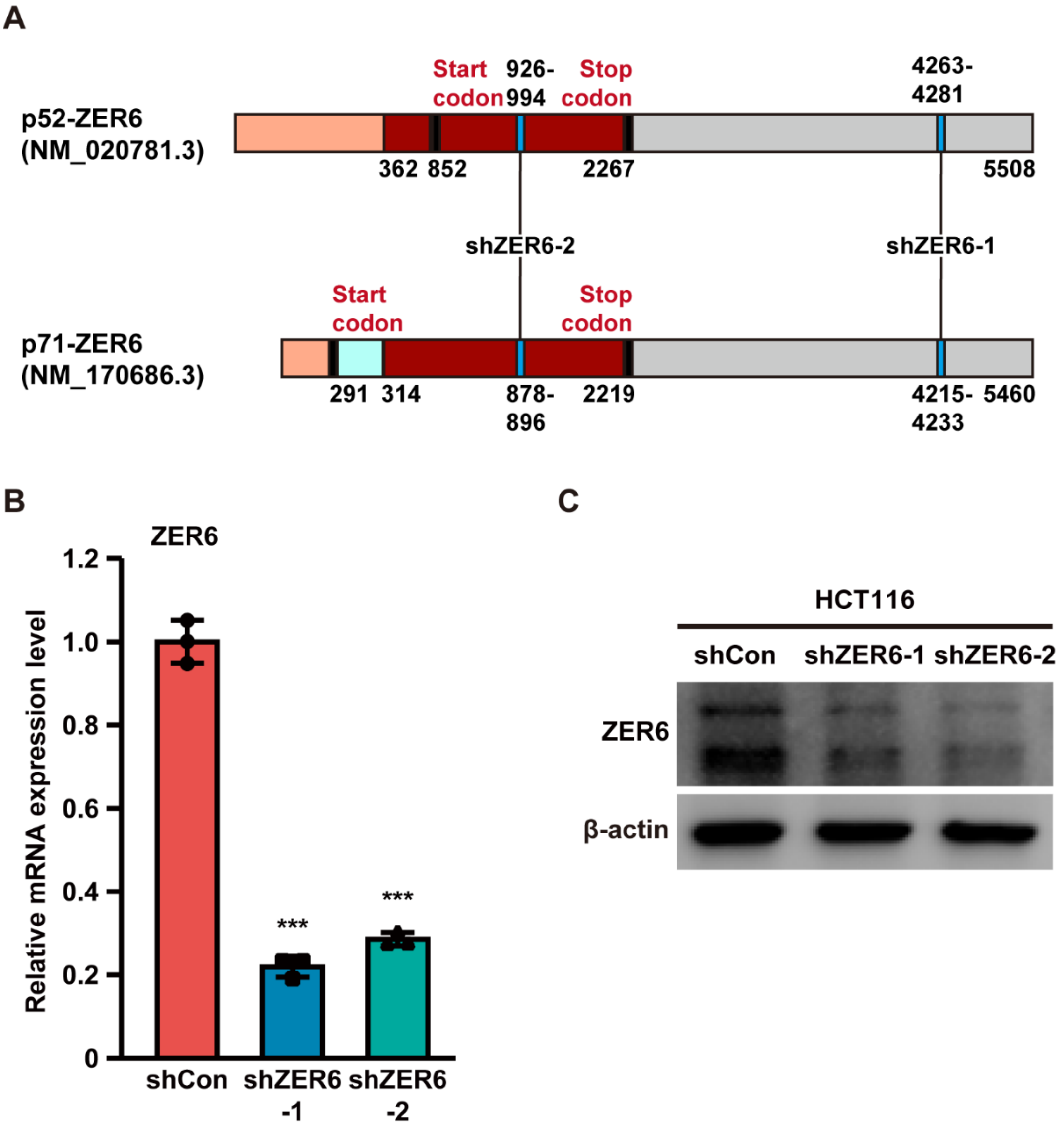

Figure S1. The efficacy of shRNA expression vectors targeting *ZER6* in HCT116 cells. A, Schematic diagram of the target sites of shRNAs targeting *ZER6*. **B,** ZER6 mRNA expression level in HCT116 cells transfected with two shRNA expression vectors targeting different sites of *ZER6*, as analyzed using quantitative real-time PCR (qRT-PCR). **C,** ZER6 protein expression level in HCT116 cells transfected with two shRNA expression vectors targeting different sites of *ZER6*, as determined using western blotting. Cells transfected with shCon were used as controls. β-actin was used for qRT-PCR normalization and as western blotting loading control. Quantification data are expressed as mean ± SD (n = 3). shZER6: shRNA expression vector targeting *ZER6*; ***$P < 0.001$.

Figure S2

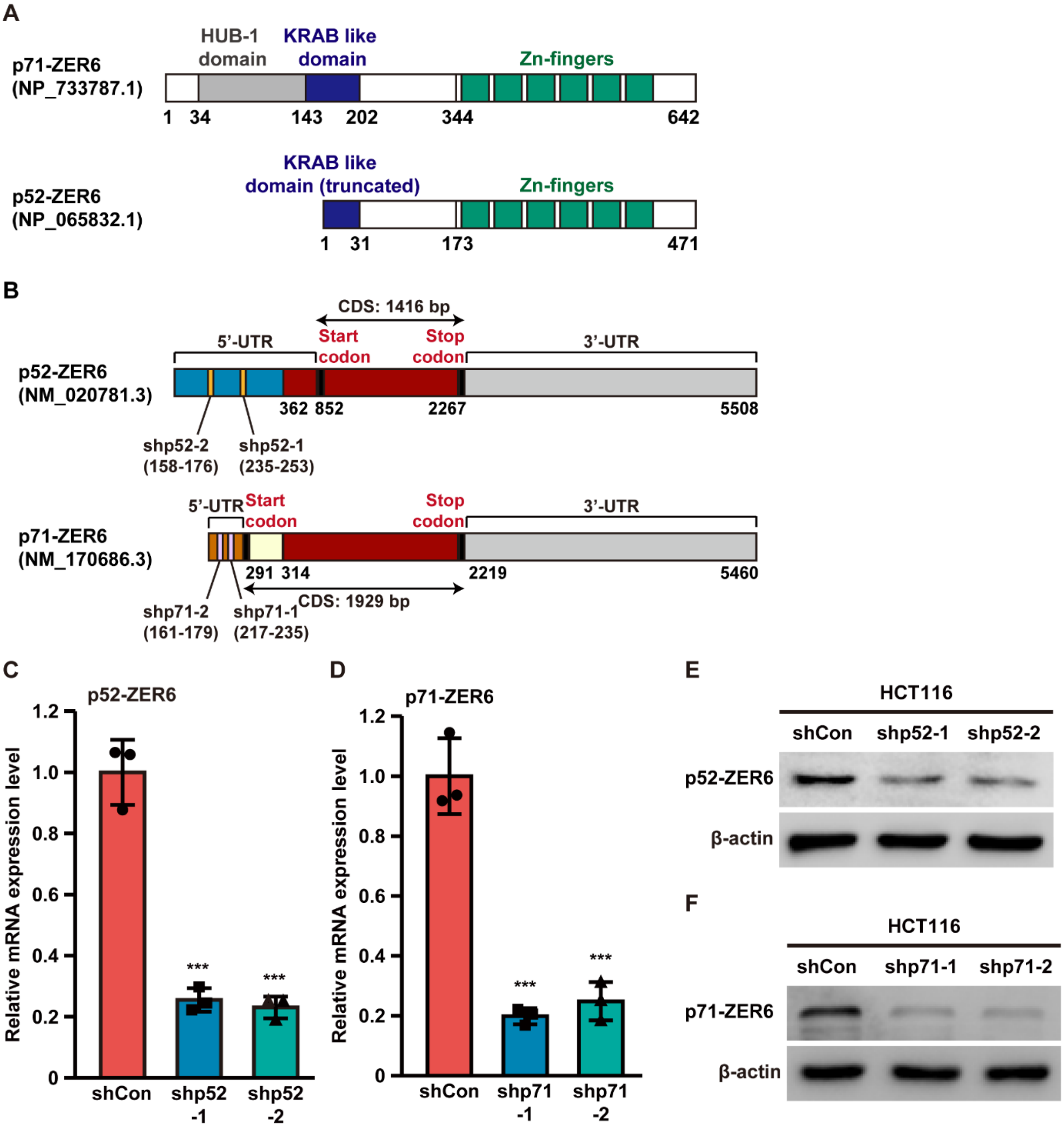

Figure S2. The efficacy of shRNA expression vectors targeting *p52-ZER6* and *p71-ZER6* in HCT116 cells. A, Schematic diagram of the amino acid sequences of ZER6 isoforms. **B,** Schematic diagram of the shRNA target sites specifically targeting *p52-ZER6* and *p71-ZER6*. **C–D,** p52-ZER6 (C) and p71-ZER6 (D) mRNA expression levels in HCT116 cells transfected with two shRNA expression vectors targeting different sites of *p52-ZER6* and *p71-ZER6*, as analyzed using qRT-PCR. **E–F,** p52-ZER6 (E) and p71-ZER6 (F) protein expression levels in HCT116 cells transfected with two shRNA expression vectors targeting different sites of *p52-ZER6* and *p71-ZER6*, as determined using western blotting. Cells transfected with shCon were used as controls. β-actin was used for qRT-PCR normalization and as western blotting loading control. Quantification data are expressed as mean ± SD (n = 3). shp52: shRNA expression vector targeting *p52-ZER6*; shp71: shRNA expression vector targeting *p71-ZER6*; $***P < 0.001$.

Figure S3

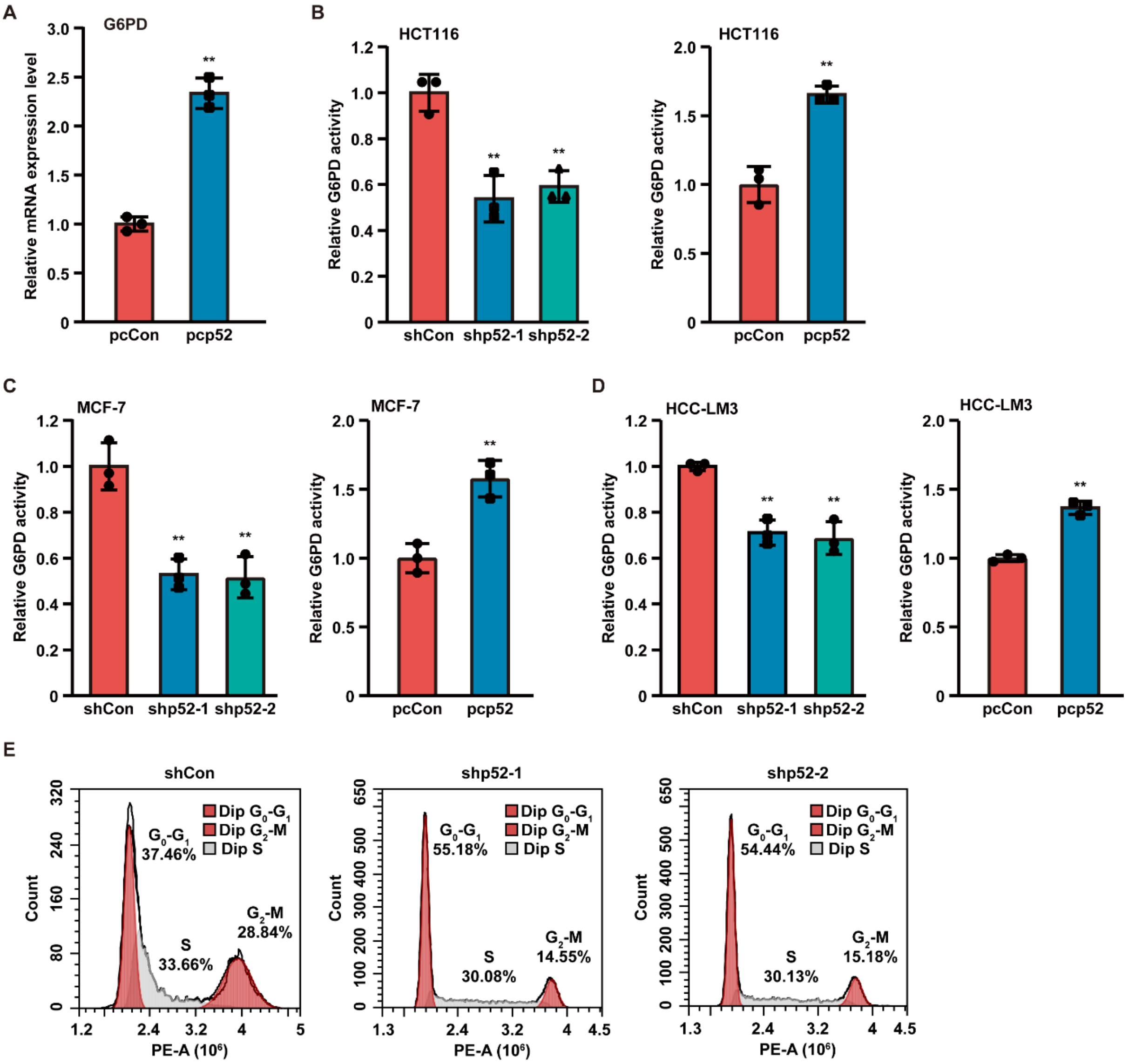

Figure S3. p52-ZER6 promotes G6PD expression and enzymatic activity. A, G6PD mRNA expression level in *p52-ZER6*-overexpressed HCT116 cells, as analyzed using qRT-PCR. **B–D,** G6PD enzymatic activities in *p52-ZER6*-silenced (left) and *p52-ZER6*-overexpressed (right) HCT116 (B), MCF-7 (C), and HCC-LM3 (D) cells. **E,** Cell cycle analysis of *p52-ZER6*-silenced HCT116 cells, as examined using PI staining and flow cytometry. Quantification data are expressed as mean ± SD (n = 3). shp52: shRNA expression vector targeting *p52-ZER6*; pcCon: pcEF9-Puro; pcp52: *p52-ZER6* overexpression vector; $**P < 0.01$.

Figure S4

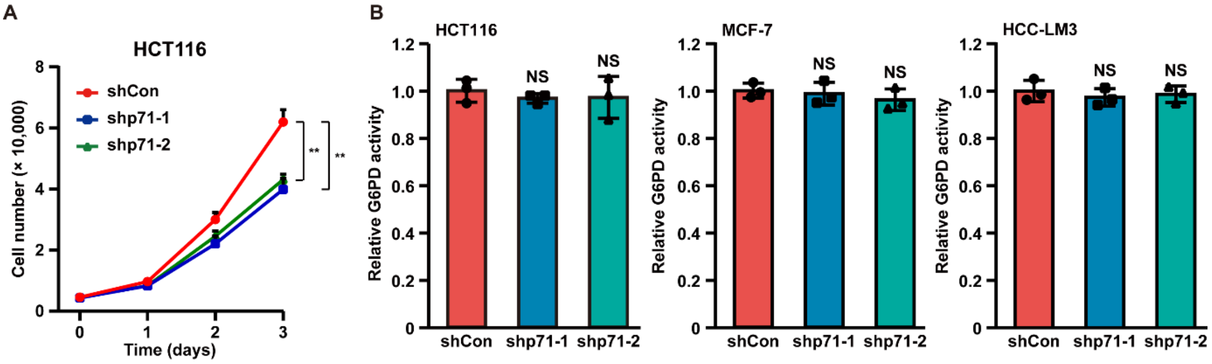

Figure S4. p71-ZER6 fails to promote G6PD enzymatic activity. A, Viability of *p71-ZER6*-silenced HCT116 cells at indicated time points. **B,** G6PD enzymatic activities in *p71-ZER6*-silenced HCT116, MCF-7, and HCC-LM3 cells. Cells transfected with shCon were used as controls. Total protein was used for normalizing the levels of G6PD enzymatic activity. Quantification data are expressed as mean ± SD (n = 3). shp71: shRNA expression vector targeting *p71-ZER6*; $^{**}P < 0.01$; NS: not significant.

Figure S5

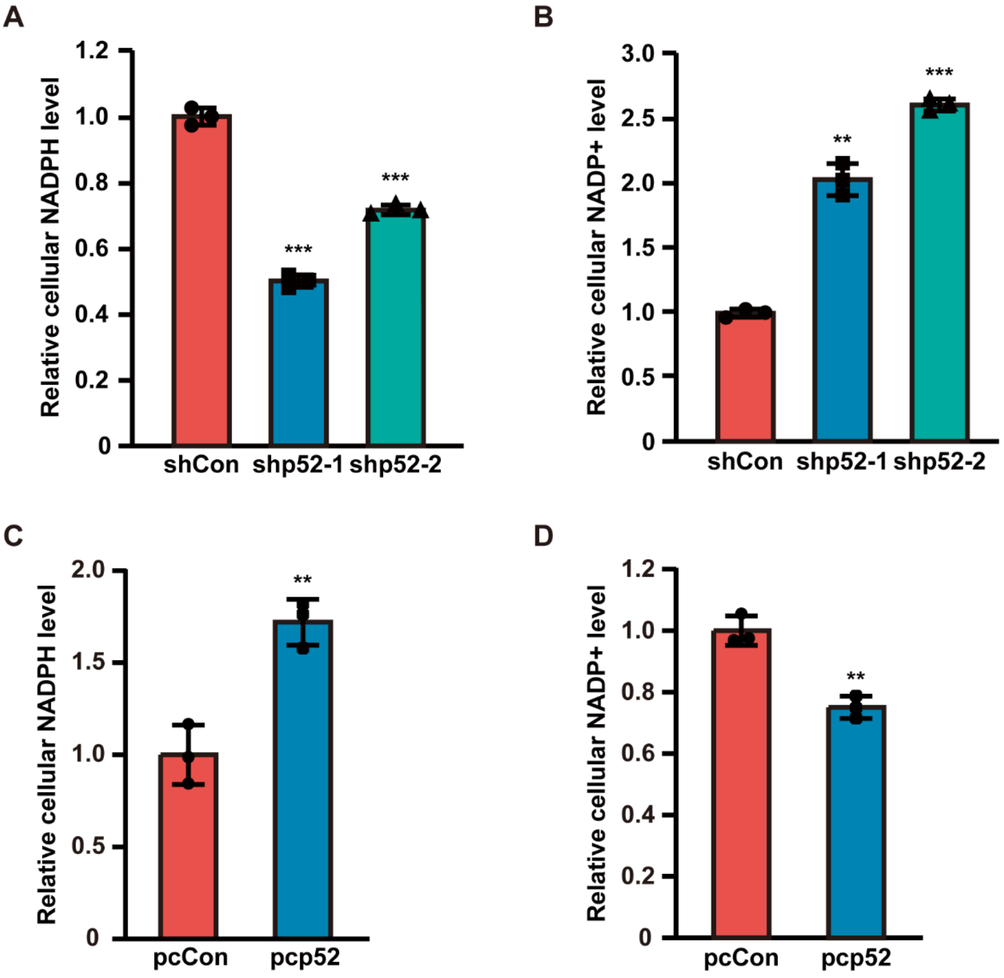

Figure S5. Effect of *p52-ZER6* alteration on intracellular NADPH and NADP$^+$ levels. A–B, Intracellular NADPH (A) and NADP$^+$ (B) levels in *p52-ZER6*-silenced HCT116 cells. **C–D,** Intracellular NADPH (C) and NADP$^+$ (D) levels in *p52-ZER6*-overexpressed HCT116 cells. Cells transfected with shCon or pcCon were used as controls. Total protein was used for normalization. Quantification data are expressed as mean ± SD (n = 3). shp52: shRNA expression vector targeting *p52-ZER6*; pcCon: pcEF9-Puro; pcp52: *p52-ZER6* overexpression vector; $^{**}P < 0.01$; $^{***}P < 0.001$.

Figure S6

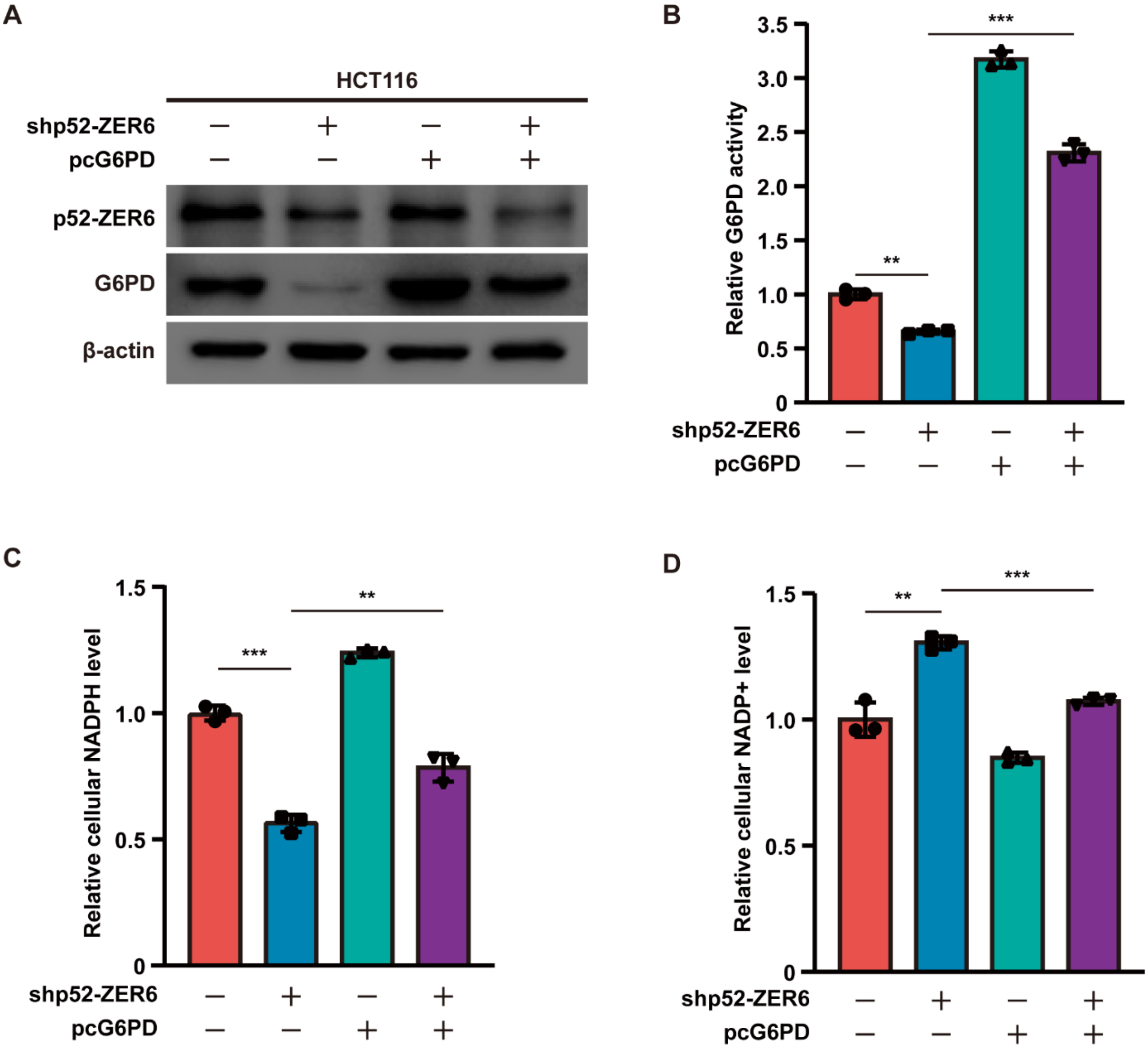

Figure S6. G6PD is crucial for p52-ZER6 regulation on cellular NADPH and NADP$^+$ levels. A, G6PD protein expression level in *p52-ZER6*-silenced, *G6PD*-overexpressed HCT116 cells, as determined using western blotting. **B,** G6PD enzymatic activity in *p52-ZER6*-silenced, *G6PD*-overexpressed HCT116 cells. **C–D,** Intracellular NADPH (C) and NADP$^+$ (D) levels in *p52-ZER6*-silenced, *G6PD*-overexpressed HCT116 cells. Cells transfected with shCon and pcCon were used as controls. β-actin was used for western blotting loading control. Total protein was used for normalizing the levels of G6PD enzymatic activity, NADPH and NADP$^+$. Quantification data are expressed as mean ± SD (n = 3). shp52: shRNA expression vector targeting *p52-ZER6*; pcCon: pcEF9-Puro; pcG6PD: *G6PD* overexpression vector; ***P* < 0.01; ****P* < 0.001.

Figure S7

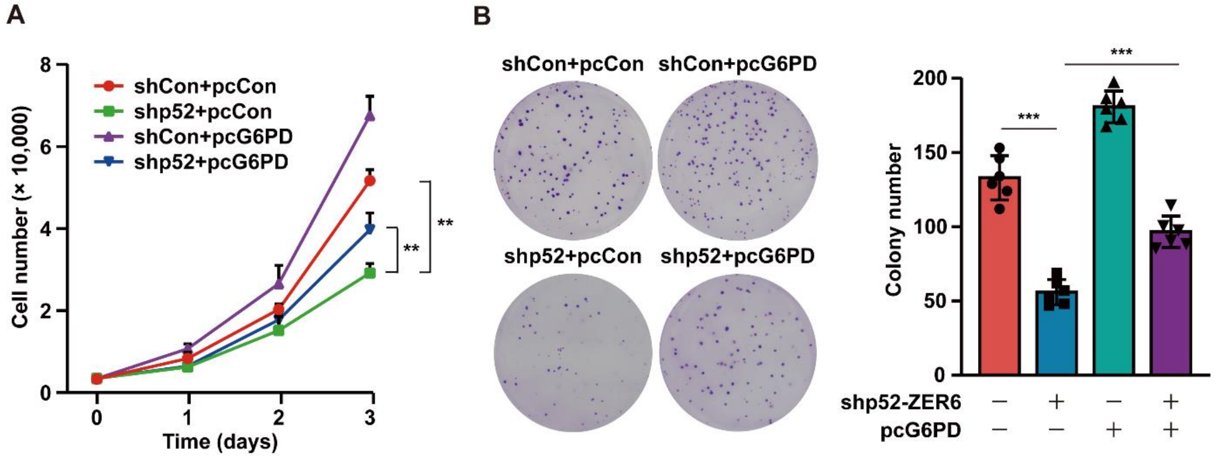

Figure S7. G6PD is crucial for p52-ZER6 oncogenic potential. A, Viability of *p52-ZER6*-silenced, *G6PD*-overexpressed HCT116 cells at indicated time points (n = 3). **B,** Colony formation potential of *p52-ZER6*-silenced, *G6PD*-overexpressed HCT116 cells. Representative images (left) and quantification results (right, n = 6) are shown. Cells transfected with shCon and pcCon were used as controls. Quantification data are expressed as mean ± SD. shp52: shRNA expression vector targeting *p52-ZER6*; pcCon: pcEF9-Puro; pcG6PD: *G6PD* overexpression vector; $^{**}P < 0.01$; $^{***}P < 0.001$.

Figure S8

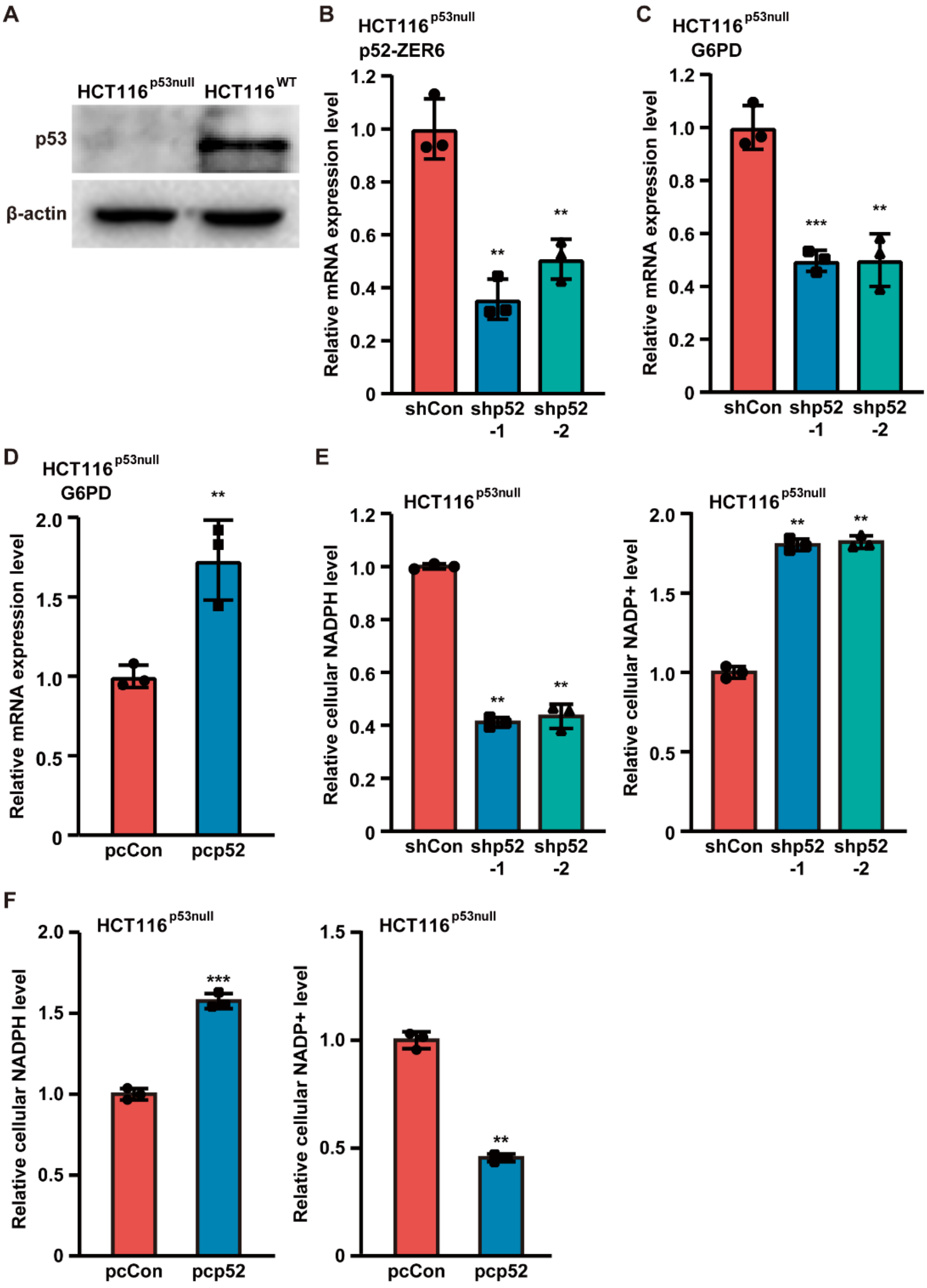

Figure S8. p52-ZER6 regulates G6PD and PPP in a p53-independent manner. A, p53 protein expression levels in HCT116^{p53null} and HCT116^{WT} cells. **B–C,** p52-ZER6 (B) and G6PD (C) mRNA expression levels in *p52-ZER6*-silenced HCT116^{p53null} cells, as analyzed using qRT-PCR. **D,** G6PD mRNA expression level in *p52-ZER6*-overexpressed HCT116^{p53null} cells, as analyzed using qRT-PCR. **E–F,** Intracellular NADPH (left) and NADP$^+$ (right) levels in *p52-ZER6*-silenced (E) and *p52-ZER6*-overexpressed (F) HCT116^{p53null} cells. Cells transfected with shCon or pcCon were used as controls. β-actin was used for qRT-PCR normalization and as western blotting loading control. Total protein was used for normalizing the levels of NADPH and NADP$^+$. Quantification data are expressed as mean ± SD (n = 3). shp52: shRNA expression vector targeting *p52-ZER6*; pcCon: pcEF9-Puro; pcp52: *p52-ZER6* overexpression vector; $^{**}P < 0.01$; $^{***}P < 0.001$.

Figure S9

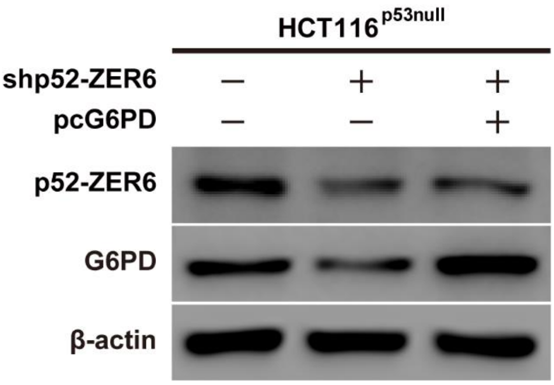

Figure S9 Establishment of *p52-ZER6*-silenced, *G6PD*-overexpressed HCT116^{p53null} stable cell line. p52-ZER6 and G6PD protein expression levels in stable cell line established from HCTT16^{p53null} transfected with shp52 and pcG6PD, as determined using western blotting. Cells transfected with shCon and pcCon were used as controls. β-actin was used as western blotting loading control. shp52: shRNA expression vector targeting *p52-ZER6*; pcCon: pcEF9-Puro; pcG6PD, *G6PD* overexpression vector.

Table S1. Primer pairs used for quantitative real time PCR.

Genes	Refseq No.	Primer sequences (5'-3')	
		Forward	Reverse
β-actin	NM_001101.5	CGAGCGCGGCTACAGCTT	TCCTTAATGTCACGCACGATTT
ZER6	Common for NM_170686.3 & NM_020781.4	CCAGAAGGGGAACATAATACAGA	CTGATGTTGAAATACCAGGCTCT
p52-ZER6	Specific for NM_020781.4	TGCGGTCGAAGACGAGAT	TCCTGCTCTCTCCATGTTCA
p71-ZER6	Specific for NM_170686.3	GTCCGTGCGGGGAAGGCA	CCATGGCCCTGCGCTGTCT
G6PD	NM_000402.4	CGTGATGCAGAACCACCTAC	TGCATTTCAACACCTTGACC
PGK1	NM_000291.4	CTGTGGCTTCTGGCATACCT	CGAGTGACAGCCTCAGCATA
TIGAR	NM_020375.3	CTCAAGACTTCGGGAAAGGA	GGTGTAAACACAGGGCACTCTT
HK2	NM_000189.5	GATGACTTCCGCACAGAATTT	TCTCACCCAGGTACATTCCAC
PKM2	NM_001411081.1	ACGTGGATGATGGGCTTATT	CCAAGGAGCCACCATTTTC
GLUT1	NM_006516.4	ACCATTGGCTCCGGTATCG	GCTCGCTCCACCACAAACA
PFK2	NM_006212.2	GCTATGAAACCAAAACCCCA	TAACGATCAGAGTCGGGGAG
LDHA	NM_005566.4	ACCCAGTTTCCACCATGATT	CCCAAAATGCAAGGAACACT
SCO2	NM_005138.3	TCGTGCTTGGTCCACTGAC	TCAGCAGCAGCATGGATCT
PDK1	NM_001278549.2	CTGTGATACGGATCAGAAACCG	TCCACCAAACAATAAAGAGTGCT
PGM1	NM_002633.3	CCGGTTCTACATGAAGGAGG	GAGGATTCCATTCTGTCCGA
FH	NM_000143.4	CGGTCAGGTCTGGGAGAAT	CCATGGTCATTGCTTCACAC
SDHC	NM_003001.5	TCAAACCGTCCTCTGTCTCC	AAGAGAGACCCCTGCACTCA

Table S2. Antibodies used for western blotting, ChIP assay, immunohistochemistry and *in situ* hybridization assay.

Antibody	Maker	Product No.	Experiment	Dilution
Anti-β-actin	Proteintech	60008-1- Ig	Western Blotting	1/10,000
Anti-ZNF398 (Anti-ZER6)	GeneTex	GTX107221	Western Blotting<br>ChIP assay<br>Immunohistochemistry	1/1,500<br>30 μg/ml cell lysate<br>1/200
Anti-G6PD	Proteintech	25413-1-AP	Western blotting<br>Immunohistochemistry	1/3,000<br>1/200
Anti-p53	Proteintech	10442-1-AP	Western blotting	1/1,000
Anti-Histone H3	Proteintech	17168-1-AP	ChIP assay	30 μg/ml cell lysate
Anti-Rabbit IgG	Proteintech	B900610	ChIP assay	30 μg/ml cell lysate
Goat Anti-Rabbit IgG	ZSGB-BIO	ZB2301	Western blotting	1/10,000
Goat Anti-Mouse IgG	ZSGB-BIO	ZB2305	Western blotting	1/10,000
Anti-DIG-HRP	Jackson ImmunoResearch	200-032-156	*in situ* hybridization assay	1/400
